# South African study of blast phase chronic myeloid leukaemia: A poor prognostic outlook

**DOI:** 10.4102/ajlm.v11i1.1578

**Published:** 2022-05-31

**Authors:** Katherine E. Hodkinson, Nikki Bouwer, Jenifer Vaughan

**Affiliations:** 1 Department of Molecular Medicine and Haematology, Faculty of Health Sciences, University of the Witwatersrand, Johannesburg, South Africa; 2 Institution of National Health Laboratory Service, Johannesburg, South Africa

**Keywords:** chronic myeloid leukaemia, blast phase, major molecular route abnormalities, South Africa, p210 *BCRABL1* fusion transcript, p190 *BCRABL1* fusion transcript, responses by quantitative reverse transcriptase-polymerase chain reaction, karyotype

## Abstract

**Background:**

Chronic myeloid leukaemia (CML) is a haematological malignancy characterised by the translocation t(9;22)(q34;q11.2), resulting in a constitutively active tyrosine kinase. Globally, overall survival of blast crisis phase (BC) CML is one year. Newer tyrosine kinase inhibitors and allogeneic stem cell transplantation offer remission; however, refractory and relapsed disease remain the biggest challenges.

**Objective:**

In South Africa, literature is lacking on BC-CML. This study aimed to determine the disease characteristics and overall survival in South Africa.

**Methods:**

This retrospective, laboratory-based study reviewed all new BC-CML diagnoses via flow cytometry at Charlotte Maxeke Johannesburg Academic Hospital in Johannesburg, South Africa, between April 2016 and October 2019. BC-CML was defined as the presence of > 20% blasts with a CML history or the *BCR-ABL1* fusion gene (p210/p190) in the appropriate clinical or pathological context. Survival outcomes were inferred from clinical and laboratory data.

**Results:**

Twenty-two new cases of BC-CML were diagnosed (median age: 34 years). There were 20 (91%) cases with the fusion transcripts p210 and two (9%) cases with p190 *BCRABL1.* For blast lineage, 14 cases were myeloid (63.6%), six were lymphoid (27.3%), and two were ambiguous (9.1%). There was a 72.7% mortality (16 cases); sepsis, refractory and relapsed disease were the major causes. Patients who achieved remission had lower blast percentages, simple karyotypes, and a trend towards higher white cell and platelet counts at presentation.

**Conclusion:**

Optimised management of early-stage CML, prevention and aggressive management of sepsis, with advocation for newer therapies are needed to improve the overall survival of BC-CML in South Africa.

## Introduction

Chronic myeloid leukaemia (CML) is a haematological malignancy that arises within a pluripotent haemopoietic stem cell.^1,2,3^ The defining molecular aberration is a translocation involving the long arms of chromosome 9 and 22 (t[9;22][q34;q11.2]) which results in a chimeric breakpoint cluster region-Abelson murine leukaemia viral oncogene homolog 1 (*BCR-ABL1*) fusion gene encoding for a constitutively active tyrosine kinase.^4,5,6,7^ This protein drives the expansion and survival of the clone through the proliferation of the myeloid compartment, anti-apoptotic properties and reduced adherence to the bone marrow niche.^8,9,10^ Chronic myeloid leukaemia is a bi- or tri-phasic disease that classically progresses through chronic, accelerated, and blast phases, as defined by the World Health Organization and European LeukaemiaNet Criteria.^11,12,13,14,15^

Tyrosine kinase inhibitors (TKIs) were the first targeted therapies developed for CML. The introduction of the first-generation TKIs as the mainstay therapy in 1998 halted the natural progression of the disease. This has allowed patients to maintain chronic phase disease with the achievement of morphological, clinical, and molecular remission targets. Since the introduction of second and third-generation TKIs, there has been advancement in both the speed and depth of molecular remission.^16^ In the United States, the overall survival (OS) in chronic phase disease is nearly equivalent to that of the general population when response milestones are achieved.^17,18^ However, disease progression to accelerated or blast phase still occurs in this era of effective targeted therapy, as a result of drug resistance, cessation of therapy resulting from drug intolerance or side effects, poor compliance, altered metabolism, and drug interactions.

In contrast, the diagnosis of chronic myeloid leukaemia in blast crises phase (BC) CML has been associated with a low median OS of 12 months in the United States. Combination therapy with TKIs and intensive chemotherapy, consolidated with an allogeneic haemopoietic stem cell transplant (AlloSCT), offers patients the most favourable outcomes.^19^ In Africa, access to second or third generation TKIs and AlloSCT is limited, and there is a paucity of data on the prevalence and outcomes of BC-CML. This study aimed to describe the disease and patient characteristics as well as the OS of BC-CML diagnosed in the state sector setting in Johannesburg, South Africa.

## Methods

### Ethical considerations

Ethics clearance was obtained from the University of the Witwatersrand Human Ethics Committee (protocol number M150160). The study was a retrospective analysis of laboratory results obtained from the laboratory information system (LIS) (TrakCare, InterSystems, Cambridge, Massachusetts, United States) and patient consent was not a requisite. Patient privacy was ensured through the use of unique study numbers, with the link to patient identifying information kept in a separate, password-protected Excel spreadsheet.

### Study design

This study included all new cases of acute leukaemia diagnosed by flow cytometry at the Charlotte Maxeke Johannesburg Academic Hospital (CMJAH) over 42 months, between 08 April 2016 and 16 October 2019. These cases were identified from the flow cytometry register with the largest proportion of cases received from CMJAH, Chris Hani Baragwanath Academic Hospital (CHBAH) and Helen Joseph Hospital. The LIS was used to collect available clinical data, demographics, laboratory results, treatment responses, OS, and cause of death. The results were collated on an Excel spreadsheet (Microsoft Corporation, Santa Rosa, California, United States) and cases of BC-CML were extracted. Inclusion of cases into the BC-CML category required a preceding history of CML or presence of the *BCR-ABL1* fusion gene (p210/ p190) in the context of splenomegaly, peripheral smear features of CML (leucocytosis, left shift, myelocyte peak, eosinophilia and basophilia) and major molecular route abnormalities on conventional cytogenetics (CCy).

### Statistical analysis

The data were analysed using STATA software, version 14.1 (Statistics/Data Analysis, StataCorp LLC, College Station, Texas, United States). Continuous data were reported as the median (interquartile ranges [IQR]) and categorical data as percentages and frequencies. The two-sample Wilcoxon rank-sum (Mann-Whitney) test was used to compare continuous variables. Statistical significance was accepted at a two-sided *p*-value of 0.05.

## Results

Twenty-two cases of BC-CML were diagnosed by flow cytometry in the reviewed period between 2016 and 2019. The rate of blastic transformation from chronic and accelerated phase CML could not be determined as the prevalence of CML was unknown for the referring centres.

### Patient demographics and treatment history

The median age was 34 years (IQR: 21 years) with two patients aged younger than 18 years, and a slight predominance of male patients, 57% (*n* = 12). HIV testing was performed in less than half (*n* = 20) of the patients with a 50% positivity (Table 1).

**TABLE 1:** Demographic and laboratory information for patients with CML in blast phase, Johannesburg, South Africa, 08 April 2016 to 16 October 2019.

Parameter	All patients (*n* = 22)	Deceased patients (*n* = 16)	Patients in remission (*n* = 5)
Male to female ratio	1.2:1	1.3:1	1.5:1
**Age (years)**			
Median	34	32	37
IQR	21	15	25
**HIV positive**			
*N*	5†	4‡	1§
%	50.0	36.0	33
**Haemoglobin (g/dL)**			
Median	7.6	7.1	7.6
IQR	3.4	4.2	0.3
**Platelet count ( × 10^9^/L)**			
Median	79	31	101
IQR	180	177	324
**White cell count ( × 10^9^/L)**			
Median	58.5	37.3	144.2
IQR	282.9	175.4	367.4
**Neutrophils ( × 10^9^/L)**			
Median	7.66	7.3	72.2
IQR	56.2	39.8	91.8
**Basophils (%)**			
Median	4.5	2. 5	5.4
IQR	11	10.5	7
**Peripheral blast count ( % )**			
Median	38.5¶	56	18
IQR	47	41	26
**Immunophenotype**			
Myeloid,			
*N*	14	9	4
%	63.6	56.3	80.0
**Lymphoid**			
*N*	6	5	1
%	27.3	31.2	20.0
**Ambiguous**			
*N*	2	2	-
%	9.1	12.5	-

Note: HIV testing was not routinely performed resulting in a the lower number of individuals with HIV results.

CML, chronic myeloid leukemia; IQR, interquartile range.

† , *N* = 10 (No HIV results available for 12 patients);

‡ , *N* = 7; (No HIV results available for 9 patients);

§ , *N* = 3 (No HIV results available for 2 patients);

¶ , *N* =21 (1 not reported).

Information on the use of TKI therapy was limited to that documented on the LIS. Imatinib mesylate therapy was reported in one patient; dasatinib in three; and nilotinib in two. None of the patients had been managed with a trial of treatment-free remission (TFR).

### Molecular and immunophenotypic results

Twenty cases had p210 *BCR-ABL1* fusion transcripts, and two cases had p190 *BCR-ABL1*. For blast lineage in BC-CML, 14 cases (63.6%) were myeloid, six cases (27.3%) were lymphoid and two cases (9.1%) were ambiguous. Of the lymphoid cases, approximately two-thirds (*n* = 4) were of B lineage. The p210 case lineages were myeloid in 13 cases; T in one case, B in four cases; and ambiguous in two cases. The p190 cases were of myeloid and T lineage, with the latter relapsing as an ambiguous lineage acute leukaemia (T/Myeloid).

**FIGURE 1:**
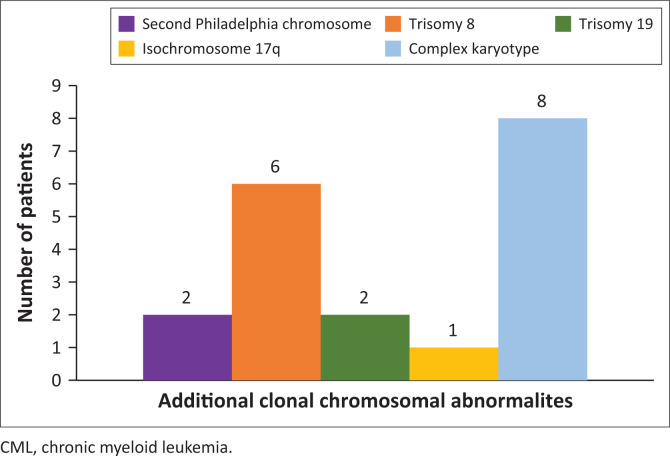
Frequency of additional clonal chromosomal abnormalities in patients with CML in blast phase (*N* = 16) Johannesburg, South Africa, 08 April 2016 to 16 October 2019. Cytogenetics analysis was available for 16 patients. Additional clonal chromosomal abnormalities was identified in 11 patients. Five of the karyotypes demonstrated multiple additional clonal chromosomal abnormalities. Trisomy 19 and isochromosome 17q were always present with a trisomy 8.

Additional clonal chromosomal abnormalities were detected on CCy in 11 of the 16 individuals with successful karyotypes. Complex karyotypes and trisomy 8 were the most frequently observed, with five CCy demonstrating multiple additional clonal chromosomal abnormalities (Figure 1). One patient bore the T315I mutation at the time of diagnosis of BC-CML with a history of treatment failure on both imatinib mesylate and nilotinib.

### Patient outcomes

**FIGURE 2:**
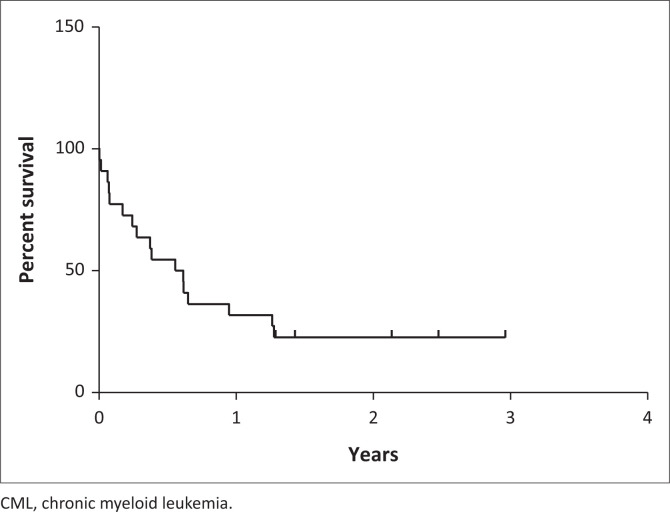
Overall survival of patients with CML in blast phase Johannesburg, South Africa, 08 April 2016 to 16 October 2019, excluding one patient, who was lost to follow-up.

There was a 72.7% (16 cases) mortality with a median OS of 7 months (IQR: 13.6 months) (Figure 2). The details of the therapeutic management of each BC-CML was not available. Five deaths occurred in the first month with sepsis the predominant cause in 80% (four cases). The remaining 11 deaths occurred at a median of 6.7 months (IQR: 4.5 months) with two-thirds of the deaths attributed to relapsed and/or refractory disease, and one-third to sepsis. Patients with the p190 *BCR-ABL1* fusion transcript survived a median of 6.1 months (IQR: 3.3 months) with relapsed disease as cause of death.

**FIGURE 3:**
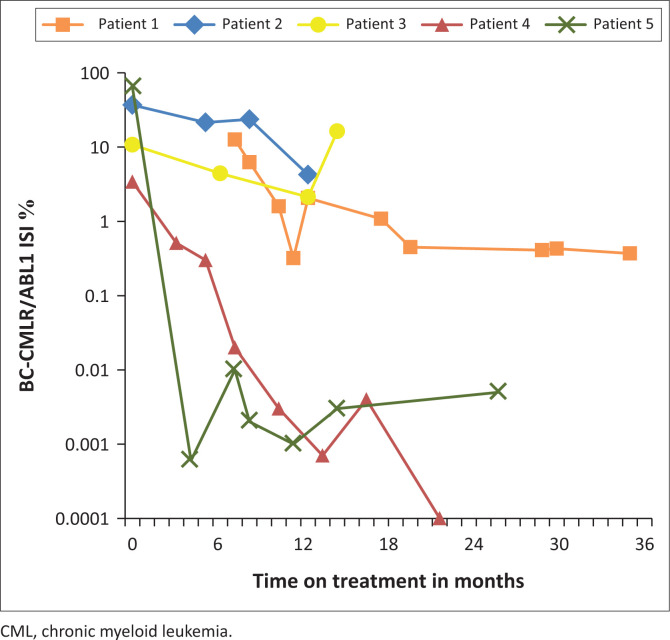
Cumulative quantitative reverse transcriptase-polymerase chain reaction results for patients in remission, from time of diagnosis of CML in blast phase (*N* = 5), Johannesburg, South Africa, 08 April 2016 to 16 October 2019. The following were the demographics of patients 1 to 5; Patient 1: 51-year-old female; Patient 2: 26-year-old HIV-positive male; Patient 3: 19-year-old HIV-negative female; Patient 4: 54-year-old male; Patient 5: 37-year-old male. All patients had a p210 BC-CMLRABL1 transcript, the blast lineage was T in patient 2 and myeloid in the remainder. Johannesburg, South Africa, 08 April 2016 to16 October 2019.

At the time of the study, remission with reversion to chronic phase CML (based on laboratory parameters) was achieved in 22.7% (five cases) with a median remission time of 25.6 months (IQR: 12.5 months). The disease characteristics in the remission group included a dominance of myeloid lineage (80%, four cases) with a second Philadelphia (Ph) chromosome being the only additional abnormality present in one of the three patients with successful CCy. The molecular responses by quantitative reverse transcriptase-polymerase chain reaction were extremely varied from undetectable transcript levels to no molecular response (Figure 3). One patient was lost to follow-up at one year with achievement of a major cytogenetic response at this time point.

The median peripheral blood blast percentage at diagnosis was significantly higher in patients who died, 56% (16 cases) versus those in remission 18% (five cases) (*p* = 0.03). Conversely, the white cell and platelet counts at diagnosis were lower among patients who died, but the difference was not statistically significant (white cell count for deceased patients: median = 37.3 (IQR: 175.4) versus for patients in remission: median, 144.2 (IQR: 367.4, *p* = 0.52) and the platelet count for deceased patients: median, 31 (IQR: 177) versus for patients in remission: median, 101 (IQR: 324, *p* = 0.54) (Table 1).

## Discussion

This retrospective laboratory report review determined the disease charatersistics and overall survival of BC-CML in South Africa. The median age of BC-CML in our study was significantly lower than the 61 years reported internationally and corresponds with the younger age at diagnosis of CML in our setting.^20,21,22^ This finding may be reflective of South African demographics, which are characterised by a young age structure of the population or alternatively this may be the result of unknown environmental exposures or unique genetic/biological characteristics. Consequently, this young age at diagnosis has a financial impact upon the public health sector, in particular for chronic phase CML. The cost resulting from the earlier initiation, and thus additional years on TKI therapy, is substantial.

The risk factors for the development of CML remain largely unknown with acute radiation exposure and rare cases of possible familial predisposition reported.^19^ To our knowledge there were no cases of familial CML in our study nor have there been published cases from South Africa.

Progression from chronic phase CML to BC-CML could not be determined from our study as a result of the paucity of local prevalence data. Indirectly, the rates of transformation at the CHBAH appear high as compared with international reports (~2%), with a yearly number of ~3 new adult cases of BC-CML, in a background of ~12 new cases of CML per year.^20,23^ Plausible reasons for higher rates of transformation include the development of TKI resistance in the initial stages of CML as a result of poor compliance or treatment interruptions, and late presentation of patients to medical facilities resulting in more advanced stage disease.

The lineage distribution of BC-CML in this study is similar to that reported in the literature with myeloid lineage accounting for around ~52% to 67% and lymphoid for around ~19% to 35%.^24,25^ The proportion of ambiguous lineage cases in our setting was similar to that seen in studies performed in Taiwan and Mexico, reported at ~12% to 14%.^24,25^ For the lymphoid lineage, an expected ratio of B to T cases was present in our study.^24,25^ This suggests a similar disease biology and accordingly supports the use of international treatment approaches/guidelines for BC-CML, with the ability to compare treatment and survival endpoints.

The p210 *BCR-ABL1* fusion (e13a1 [b2a2]/e14a2 [b3a2]) is the most common transcript seen in CML. Co-expression of p210 with p190 (e1a2) is described, but a very rare phenomenon. Rarer still is an isolated p190 *BCR-ABL1* fusion which has been described in 1% of CML. This latter group is associated with poor response to TKI therapy and disease progression in greater than 80% of the group.^26^ These characteristics likely explain the higher than expected number of p190 BC-CML cases in this study (9.1%). The median (IQR) OS in these cases is reported at ~13 months (8–40), a figure which is double that seen in our study. The reason for this difference likely reflects a lack of alternative or novel treatment options for relapsed/refractory disease in the South African public health sector.^27^ Given the high risk of this subgroup of patients, close monitoring of the therapeutic response is imperative (especially during the early phases of CML), with early intervention should the response be lost.^26^

High-risk cytogenetic abnormalities in CML include a complex karyotype and major molecular route abnormalities which are markers of disease acceleration. This is associated with more rapid disease progression, treatment resistance or suboptimal treatment response.^28^ The major molecular route abnormalities include a second Philadelphia chromosome, isochromosome I (17q), trisomy 8, and 19.^29^ Unsurprisingly, a high percentage of the BC-CML cases with existing CCy bore these abnormalities. As a result of the low number of cases in remission and with CCy, correlations between molecular characteristics and outcomes could not be made. However, a second Philadelphia chromosome was the only additional chromosomal abnormality present. Notably, in ~30% of cases, no sample was submitted for CCy or the procedure was unsuccessful. CCy is critical to prognostication, detection of clonal evolution, monitoring treatment response and directing therapeutic decisions. Practically, in such cases, peripheral blood may serve as an easily accessible, alternative source for CCy. Cognisance of this would help to limit the number of cases missing valuable molecular information. Notably, but not unexpectedly, alternative markers of high-risk CML were present in this cohort; these included peripheral blood blast percentages of > 10%, p190 *BCR-ABL1* transcripts and platelet counts of < 102 × 10^^9^/L.^19,30^

The TKIs for the treatment of chronic phase CML at the time of this study included: the first-generation TKI, imatinib mesylate and second-generation TKI, dasatinib (on a compassionate basis) and nilotinib. Currently, in the South African state sector, imatinib mesylate remains the first-line therapy together with hydroxyurea (for cytoreduction) in chronic phase CML. Nilotinib is the second line TKI and is used in situations of treatment failure. Dasatinib is no longer available on compassionate grounds. Importantly, comparable response rates and survival outcomes have been demonstrated for these second-generation TKIs.^31^ Despite a minimal risk of worsening renal dysfunction and bleeding in those with pre-existing renal dysfunction, treated with nilotinib or dasatinib, both agents can be used in the majority of patients with mild to moderate renal dysfunction as well as those with mild liver dysfunction.^32^ In the situation of a T315I mutation or treatment resistance to two sequential TKIs, motivation can be made for the potent third-generation TKI, ponatinib. With life expectancies nearing that of the general population and the associated chronicity of treatment, the practice of TFR was introduced. This refers to the discontinuation of TKI therapy with a durable off-treatment molecular response. Treatment-free remission aims to limit drug toxicities and adverse effects, reduce the financial burden of therapy and prevent teratogenicity. Several guidelines have been developed to identify patients eligible for TFR; the criteria include age ≥ 18 years, the presence of typical *BCR-ABL1* transcripts, TKI therapy used for a minimum of five years, the achievement of a deep molecular response (DMR) ≤ 4.5, a sustained DMR of ≤ 4–4.5 for ≥ 2–3 years, no prior history of accelerated or BC-CML and the ability to perform frequent (monthly initially) responses by quantitative reverse transcriptase-polymerase chain reaction testing which is both sensitive (minimum MR 4.5) and delivered within a rapid turnaround time.^33,34,35^ Given these requirements, TFR is not commonplace in the South African public sector, because it requires frequent follow-ups and timeous molecular results. The reported success of TFR is about 40% to 60%, with the possibility to regain a DMR in those who fail if intervention is prompt.^36^ Thus future studies should aim to identify factors that can predict a successful TFR.

The median OS in our cohort was almost half of that reported in a United States-based study (7 vs 12 months), driven by a substantial proportion of patients with early deaths (defined as deaths within the first month of diagnosis).^19^ These were predominantly sepsis-related, highlighting the need for improved and optimised supportive care for these patients. In our setting, BC-CML is treated with high dose imatinib mesylate and combination chemotherapy, the latter determined by the blast lineage.^37^ Additionally, intrathecal chemotherapy is initiated in patients with lymphoid BC-CML. At the time of the study, AlloSCT was not routinely available at any of the treatment centres in this cohort, further driving the poor outcomes.^38,39,40,41^ Reports suggest that AlloSCT gives the best chance of long-term remission. However, in a minority, there could be a return to pre-transplantation chronic phase disease.^37,42^ Globally, the outcomes of BC-CML are poor and novel treatment approaches are needed. Experience in the United States with dasatinib monotherapy showed a median OS of 11.8 months in myeloid and and 5.3 months in lymphoid BC-CML.^43^ The combination of hyper-CVAD (hyperfractionated cyclophosphamide, vincristine sulfate, doxorubicin hydrochloride, and dexamethasone) with dasatinib in lymphoid BC-CML resulted in a 5-year OS rate approaching 60%, which was further improved to 88% when followed by AlloSCT in the first remission.^40,44^ Use of the selective BCL2 inhibitor venetoclax has shown synergistic effect when used in combination with dasatinib in heavily pre-treated patients with BC-CML (myeloid), with a median OS of 10.9 months (with particular benefit noted in those with a dominant *BC-CMLR-ABL1* clone).^45^ Unfortunately, these novel agents and combination therapies are not available within the South African state sector and AlloSCT is not uniformly accessible. There is a great need to advocate for these new and novel approaches to improve the currently dismal outcome of this disease in our setting.^37^

A separate challenge faced for clinicians in the management of CML is the need to screen and monitor for the development of cardovascular events and second malignancies.^46^ There is a significantly increased incidence of cardiovascular and arteriothrombotic events (incidence ratio 95% confidence interval: for cardiovascular of 8.6 [7.6–9.8], and for arteriothrombotic of 1.7 [1.2–2.2]) in patients using second and third-generation TKIs (except bosutinib) and in individuals with pre-existing cardiovascular risk factors.^46,47^ In regard to second malignancies, a large United States-based study reported a slightly increased risk of CML (overall standardised incidence ratio of 1.204, 95% confidence interval: 1.108–1.301, except breast cancer) and a higher risk of death in this group.^44^ The most common sites of involvement included the digestive, skin, and urinary systems. The reason for this increased incidence is unknown but may reflect a patient-specific risk of malignancy (genetic/exposure).^46^ Given these findings it is imperative to identify and aggressively manage cardiovascular risk factors at baseline to prevent morbidity and mortality. Screening for second malignancies poses more of a challenge as a result of the varied sites of potential involvement and the costs involved in routine radiological screening and endoscopies. Realistically in our setting, the clinical history and findings are used as a screening tool to identify patients that require further work-up for second malignancies.

At present, the most effective management of BC-CML is its prevention. This is through the achievement of rapid and deeper molecular responses in chronic and accelerated disease, identification of high-risk individuals with close monitoring (laboratory, clinical) and timeous therapy changes in non-responders. More advocay for new and novel treatment approaches in our setting is necessary.

### Limitations

The limitations in this study include the paucity of clinical information and reliable outcome data as a result of the retrospective design. Cases without flow cytometry, especially isolated extramedullary transformation, were missed. Furthermore, molecular information, namely CCy or responses by quantitative reverse transcriptase-polymerase chain reaction, was not available for a notable proportion of the patients, preventing an accurate picture of the molecular landscape of BC-CML. Lastly, given the rarity of this entity, factors which affect outcomes are difficult to determine.

### Conclusion

A laboratory-based retrospective study of BC-CML was performed in a public health sector setting in South Africa. The results showed that BC-CML occurs at a younger age in adults as compared with international studies. This corresponds to the younger age of chronic phase CML seen in our setting, a finding which may reflect the country demographics, different environmental exposures or genetic predispositions. Disease characteristics were similar when compared with other reports of BC-CML, supporting the use of international treatment protocols and guidelines. There was a dismal overall survival of 7 months, with sepsis and relapsed refractory disease being the major contributors, highlighting the need to advocate for new and novel therapies.
